# The impact of photodynamic therapy on cellular immune function in patients with cervical HPV infection

**DOI:** 10.1016/j.clinsp.2024.100537

**Published:** 2024-12-07

**Authors:** Yan Ju, Qunyan Zhou

**Affiliations:** a Department of Gynecology, Zhejiang Medical & Health Group Hangzhou Hospital, Hangzhou 310022, Zhejiang, China; b Department of Gynecology, Hangzhou Geriatric Hospital, Hangzhou, 310022, Zhejiang, China

**Keywords:** Photodynamic therapy, Cervical HPV infection, Cellular immune function, T-cell subsets, Cytokines

## Abstract

**Introduction:**

Cervical Human Papillomavirus (HPV) infection, a common sexually transmitted infection, can lead to cervical cancer. This study investigates the impact of Photodynamic Therapy (PDT) on cellular immune function in cervical HPV-infected patients.

**Methods:**

From October 2022 to February 2023, 60 patients with cervical HPV infection were randomly assigned to a control group (conventional treatment) or a PDT group (PDT treatment), each with 30 patients. Peripheral blood samples and cervical tissue were collected before treatment, at 3 months, and at 6 months post-treatment. Flow cytometry assessed T-cell subsets (CD4^+^, CD8^+^, CD3^+^), and ELISA measured cytokine levels (TNF-α, IL-6, IL-8).

**Results:**

Baseline characteristics were similar between the PDT and control groups. The PDT group showed significantly higher HPV clearance rates at 3-months (70.00 %) and 6 months (100.00 %) compared to the control group 43.33 % and 80.00 %, respectively (p < 0.05). PDT significantly increased CD3^+^ and CD4^+^ cell levels at 3- and 6-months post-treatment compared to controls (p < 0.05). CD8^+^ levels and the CD4^+^/CD8^+^ ratio also significantly increased in the PDT group at 6 months. Additionally, PDT significantly reduced IL-6, IL-8, and TNF-α levels at 3- and 6-months post-treatment (p < 0.05).

**Conclusion:**

PDT enhances cellular immune function in cervical HPV-infected patients by increasing CD4^+^ and CD8^+^ T-cells and reducing pro-inflammatory cytokines. These findings highlight the potential of PDT in modulating immune responses and improving therapeutic strategies for cervical HPV infection.

## Highlights

• Photodynamic Therapy (PDT) significantly increased HPV clearance rates in cervical HPV-infected patients.
• PDT treatment augmented CD3+ and CD4+ T-cell levels, with sustained elevation observed up to 6 months post-treatment.
• A higher CD4+/CD8+ ratio was noted in the PDT group, indicating an enhanced cellular immune response.
• Pro-inflammatory cytokines IL-6, IL-8, and TNF-α levels decreased post-PDT, suggesting reduced inflammation.

## Background

Cervical Human Papillomavirus (HPV) infection is a common sexually transmitted infection that can lead to the development of cervical cancer.^1^ Photodynamic Therapy (PDT) has emerged as a promising treatment modality for cervical HPV infection.^2^ However, the impact of PDT on cellular immune function in patients with cervical HPV infection remains unclear. This study aims to investigate the effects of PDT on cellular immune function in patients with cervical HPV infection. HPV infection is a major contributing factor to the development of cervical cancer, with high incidence rates globally.^3^ Although progress has been made in vaccination and routine screening, many patients still do not have access to timely and effective treatment. Therefore, the search for new treatment modalities is of great significance.

PDT is a treatment approach based on photosensitizers and light exposure and has been widely used in various cancer therapies.^4^^,^^5^ In PDT, photosensitizers are administered to patients either systemically or topically, followed by light irradiation to activate the generated reactive oxygen species, which can kill or inhibit tumor cell growth.^6^^,^^7^ Recent studies have shown promising results of PDT in HPV-related cervical lesions,^7^, ^8^, ^9^ but its impact on cellular immune function remains unclear. Cellular immune function plays a crucial role in controlling and clearing pathogen infections and inhibiting tumor growth. Disruptions in cellular immune function in cervical HPV infection may contribute to persistent viral infection and progression of lesions. Therefore, understanding the effects of PDT on cellular immune function in patients with cervical HPV infection is of paramount importance for optimizing treatment strategies.

This study aims to comprehensively evaluate the impact of PDT on cellular immune function in patients with cervical HPV infection, shedding light on its role in modulating immune responses. By assessing changes in T-cell subsets, cytokine levels, and other relevant markers, the authors will explore the underlying mechanisms through which PDT regulates immune function in patients with cervical HPV infection, providing important theoretical evidence for clinical treatment. In light of this background, the authors will conduct this study to further elucidate the effects of PDT on cellular immune function in patients with cervical HPV infection, with the aim of providing new insights and approaches to improve treatment strategies for cervical cancer.

## Materials and methods

### Study participants

This study was designed as a longitudinal prospective cohort investigation. A total of 60 diagnosed with cervical HPV infection between October 2022 and February 2023 were enrolled in the study and randomly assigned to either the control group (n = 30) or the treatment group (n = 30). After taking into account factors such as missing follow-up, a total of 100 patients diagnosed with cervical HPV infection were enrolled in the study. The sample size was calculated using PASS software, with a test power of 0.8 and a test level of 0.05. Inclusion criteria: 1) The external genitalia exhibit papillary and vegetative neoplasms, all of which are new cases. 2) Cervical HPV screening reveals positive results for the following high-risk HPV types: 16, 18, 31, 33, 35, 39, 45, 51, 52, 53, 56, 58, 59, 66 or 68 either as a single type or in combination with others; this positivity persists for at least one year. 3) Patients and their families possess comprehensive knowledge regarding the study's content and willingly participate by signing the informed consent form. Exclusion criteria: 1) Individuals allergic to 5-Aminolevulinic Acid (ALA) or sensitive to light (photosensitivity). 2) Pregnant or lactating women. 3) Participants with syphilis or HIV infection. 4) Individuals already diagnosed with cervical cancer or other malignancies of the reproductive system. 5) Participants with severe autoimmune diseases, diabetes, or those receiving systemic corticosteroid therapy. Prior to treatment, a comprehensive assessment was performed to exclude any contraindications for PDT. Exclusion criteria included a history of other medications or physical treatments targeting cervical HPV infection within the past 3-months, as well as the use of systemic immunosuppressive therapy. The application for this study was submitted to the hospital's Ethics Committee and commenced upon approval (Approval n° 2022007). The patients' families signed an informed consent form. The procedures of this study followed the CONSORT Statement.

### Research methodology

After enrollment, all patients underwent colposcopy for assistance. Cervical exfoliative cell tests were performed, and HC-2 tests were conducted before treatment, during the 3-month treatment period, and during the 6-month follow-up. Firstly, secretions from the patient's cervical opening were removed. The cervical sampler was then rotated about 5 times in a consistent direction at the cervical opening and placed into the prepared preservation solution. Subsequently, cell lysis, hybridization, capture, signal amplification, and detection procedures were carried out to determine 13 common clinical HPV subtypes. In the control group, conventional microwave therapy was administered by routinely disinfecting the vagina vulva, and cervix using a microwave instrument with a power of 35 W. The microwave probe was radially burned close to the erosive surface. Patients in the treatment group received PDT (Photodynamic Therapy). The timing of treatment was carefully selected within 3‒7 days after menstruation ended while sexual activity was prohibited following menstruation. The PDT procedure followed a standardized protocol that included thorough pre-preparation. A photon cold gel containing 20 % 5-aminolevulinic acid was evenly applied externally on the surface of the cervix, cervical canal, and vaginal mucosa, using a total volume of 3 mL. The vaginal opening was then sealed, and the gel was left in place for a duration of 3.5‒4 h. Subsequently, photodynamic therapy was conducted using a photodynamic laser treatment device emitting light at a wavelength of 635 nm. The cervix and vagina were irradiated for 30 min each, with a maximum output power of 150 mW. The total energy delivered during the laser irradiation was adjusted to achieve a range of (60‒100 J/cm^2^).

### Cellular immune function analysis

The subjects were subjected to cervical tissue and morning fasting peripheral blood collection. Quantitative detection of T lymphocytes (CD3^+^, CD4^+^, CD8^+^) was performed using flow cytometry (Beckman Coulter, CytoFLEX S, USA). The expression levels of CD3^+^, CD4^+^ and CD8^+^ in both cervical tissue and peripheral blood were observed. The peripheral blood sample was collected by drawing 1.5 ml into EDTA tubes and subsequently incubated with fixation buffer (BD Biosciences, San Jose, USA) for 15 min at room temperature. The specific test kits utilized for peripheral blood cytokine levels (IL-6, IL-8, and TNF-α) were sourced from Beyotime (PI330 for Human IL-6 ELISA Kit, PI640 for Human IL-8 ELISA Kit, and PT518 for Human TNF-α ELISA Kit). Subsequently, the patients underwent a single course of PDT consisting of three treatment sessions administered once a week. Post-treatment follow-up evaluations were conducted at 3 months and 6 months after the initial PDT session. Additionally, cervical HPV testing was performed 3‒6 months postoperatively to evaluate the HPV clearance rate. Statistical analysis was then employed to analyze the collected data.

### Statistical analysis

The clinical data of 60 patients with cervical HPV infection included in this study were processed and analyzed using SPSS 26.0 statistical software. The measurement data were analyzed using a *t*-test (χ±s), while the counting data were analyzed using a χ^2^ test ( %, n). Statistical significance was determined when p < 0.05.

## Results

### Clinical characteristics of study participants

The age, education level, occupational status, economic status, reproductive history, family history, smoking history, drinking history and sexual history showed no statistically significant differences between the two groups (p < 0.05). The information can be found in Table 1.

**Table 1:** Clinical characteristics of study participants [n (%)/(χ ± s)].

Characteristic / Group	PDT Group (n = 30)	Control Group (n = 30)	χ^2^/*t*-value	p-value
Age (years)	35.46 ± 2.67	34.24 ± 2.83	1.717	0.091
Education Level			0.883	0.347
High school or below	5 (16.67)	8 (26.67)		
College or above	25 (83.33)	22 (73.33)		
Occupational Status			0.317	0.573
Employed	20 (66.67)	22 (73.33)		
Unemployed/Retired	10 (33.33)	8 (26.67)		
Marital Status			0.287	0.592
Married	20 (66.67)	18 (60.00)		
Single/Divorced	10 (33.33)	12 (40.00)		
Economic Status			1.714	0.190
≤ $5,000	10 (33.33)	15 (50.00)		
> $5,000	20 (66.67)	15 (50.00)		
Fertility History			0.287	0.592
Fertile	20 (66.67)	18 (60.00)		
Infertile	10 (33.33)	12 (40.00)		
Family History of Disease			0.287	0.592
Yes	10 (33.33)	12 (40.00)		
No	20 (66.67)	18 (60.00)		
Smoking Status			0.067	0.796
Smoker	15 (50.00)	14 (46.67)		
Non-smoker	15 (50.00)	16 (53.33)		
Alcohol Consumption			0.077	0.781
Consumer	10 (33.33)	9 (30.00)		
Non-consumer	20 (66.67)	21 (70.00)		
Sexual History			0.287	0.592
Multiple partners	10 (33.33)	12 (40.00)		
Single partner	20 (66.67)	18 (60.00)		

### The distribution patterns of subtypes of cervical HPV infection

**Fig. 1:**
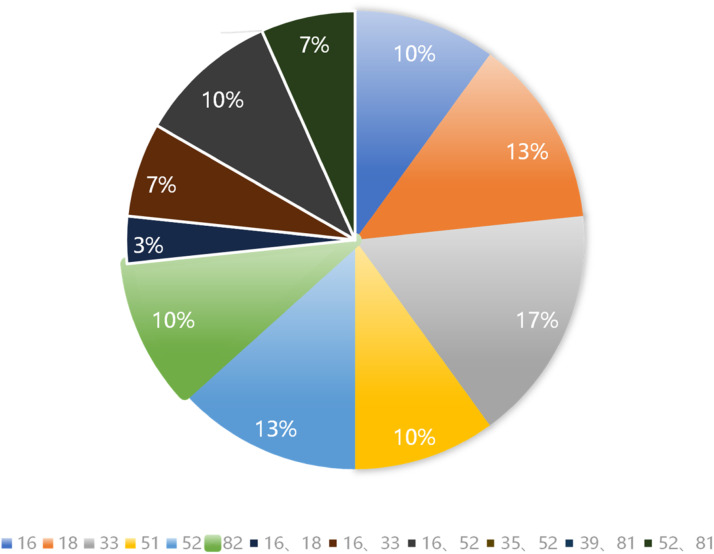
HPV infection status of patients before treatment.

After the detection of HC-2, a total of 12 types of infections were identified among 60 patients, which provides a representative sample of the population. The cervical HPV infection type showed no statistically significant difference between the control group and the treatment group (p > 0.05). The details can be found in Fig. 1 and Table 2.

**Table 2:** The distribution of HPV infection types in study groups [n (%)].

HPV Infection Types	PDT Group (n = 30)	Control Group (n = 30)	*χ^2^*-value	p-value
16	3 (10.00)	3 (10.00)	0	1
18	4 (13.33)	3 (10.00)	0.162	0.688
33	5 (16.67)	3 (10.00)	0.577	0.448
51	3 (10.00)	4 (13.33)	0.162	0.688
52	4 (13.33)	4 (13.33)	0	1
82	3 (10.00)	4 (13.33)	0.162	0.688
16, 18	1 (3.33)	3 (10.00)	1.071	0.301
16, 33	2 (6.67)	0	2.069	0.150
16, 52	3 (10.00)	2 (6.67)	0.208	0.640
35, 52	0	1 (3.33)	1.017	0.313
39, 81	0	1 (3.33)	1.017	0.313
52, 81	2 (6.67)	2 (6.67)	0	1

### HPV test results and clearance of HPV virus in various cohorts

The HPV virus clearance rate of the treatment group at 3 months and 6 months was 70.00 % and 100.00 %, respectively, which were significantly higher than those of the control group at 43.33 % and 80.00 % (p < 0.05). The information can be found in Table 3.

**Table 3:** HPV test results and clearance of HPV virus in various cohorts [n (%)].

Group	Treatment for 3-months		Treatment for 6-months	
	HPV testing negative	HPV virus clearance rate (%)	HPV testing negative	HPV virus clearance rate ( %)
Control Group (n = 30)	13	43.33	24	80.00
PDT Group (n = 30)	21	70.00	30	100.00
χ^2^-value	4.344		4.630	
p-value	0.037		0.031	

### Evaluation of cellular immune function

Table 4 presents the alterations observed in the counts of CD3^+^, CD4^+^, and CD8^+^ cells, as well as in the CD4^+^/CD8^+^ ratio identified within cervical tissues. Before treatment, there were no significant differences observed in the CD3^+^, CD4^+^, CD8^+^, and CD4^+^/CD8^+^ levels between the PDT treatment group and the control group (p > 0.05). At 3 months after treatment, the CD3^+^ and CD4^+^ levels in the PDT treatment group showed a significant increase compared to the control group (p < 0.05). However, there was no significant difference in the CD8^+^ levels between the two groups at this time point (p > 0.05). By 6 months after treatment, the PDT treatment group exhibited significantly higher CD3^+^, CD4^+^, and CD8^+^ levels compared to the control group (p < 0.05). Moreover, the CD4^+^/CD8^+^ ratio in the PDT treatment group was notably elevated compared to the control group (p<0.05). These findings suggest that PDT treatment effectively modulates T-lymphocyte subset expression in cervical local tissues, contributing to immunomodulatory effects that may play a crucial role in therapeutic outcomes. In Table 5 presents the alterations observed in the counts of CD3^+^, CD4^+^, and CD8^+^ cells, as well as in the CD4^+^/CD8^+^ ratio identified within peripheral blood. Before treatment, there were no significant differences between the two groups in CD3^+^, CD4^+^, CD8^+^, and CD4^+^/CD8^+^ levels (p > 0.05). However, noteworthy changes were observed after treatment. At 3 months post-treatment, the PDT treatment group showed significantly higher levels of CD3^+^ and CD4^+^ compared to the control group (p < 0.05), while CD8^+^ levels did not exhibit significant differences (p > 0.05). By the 6^th^ month post-treatment, the PDT treatment group demonstrated significantly elevated CD3^+^, CD4^+^, and CD8^+^ levels compared to the control group (p < 0.05). Additionally, the CD4^+^/CD8^+^ ratio in the PDT treatment group significantly increased (p < 0.05). These findings suggest that Photodynamic Therapy (PDT) can effectively modulate the expression of T-lymphocyte subsets in patients with cervical HPV infection, thereby contributing to immunomodulatory effects that may have a significant impact on treatment outcomes. In Table 4, Table 5, the authors compared markers associated with peripheral blood T-lymphocyte subsets.

**Table 4:** Comparison of T lymphocyte subsets expression in cervical local tissues between PDT treatment group and control group (χ ± s).

Indicator	Control Group	PDT treatment group	*t*-value	p-value
CD3^+^ (%)				
Before treatment	48.56 ± 3.25	48.60 ± 2.01	0.057	0.954
3-months after treatment	50.48 ± 2.39^a^	53.17 ± 1.87^a^	4.855	<0.001
6-months after treatment	49.98 ± 1.36^a^^,^^b^	58.41 ± 1.58^a^^,^^b^	22.148	<0.001
CD4^+^ (%)				
Before treatment	19.56 ± 1.04	19.58 ± 1.36	0.064	0.949
3-months after treatment	19.53 ± 1.47^a^	21.56 ± 2.15^a^	4.269	<0.001
6-months after treatment	20.85 ± 2.54^a^^,^^b^	26.48 ± 1.69^a^^,^^b^	10.108	<0.001
CD8^+^ (%)				
Before treatment	21.58 ± 1.64	21.60 ± 2.01	0.042	0.966
3-months after treatment	20.58 ± 1.47^a^	23.39 ± 1.58^a^	7.132	<0.001
6-months after treatment	22.56 ± 4.18^a^^,^^b^	26.24 ± 1.02^a^^,^^b^	4.685	<0.001
CD4^+^/CD8^+^				
Before treatment	0.91 ± 0.27	0.90 ± 0.36	0.122	0.904
3-months after treatment	0.94 ± 0.15^a^	0.92 ± 0.45^a^	0.231	0.818
6-months after treatment	0.88 ± 0.14^a^^,^^b^	1.03 ± 0.25^a^^,^^b^	2.867	0.006

Note: Compared with before treatment.

a p < 0.05; Compared with 3 months after treatment,

b p < 0.05.

**Table 5:** Changes in peripheral blood T-lymphocyte subsets markers after PDT treatment (χ ± s).

Indicator	Control Group	PDT treatment group	*t*-value	p-value
CD3^+^ (%)				
Before treatment	32.5 ± 2.11	31.8 ± 2.05	1.303	0.198
3 months after treatment	31.8 ± 2.03^a^	31.4 ± 1.98^a^	0.773	0.443
6 months after treatment	30.6 ± 1.97^a^^,^^b^	35.8 ± 1.78^a^^,^^b^	10.727	<0.001
CD4^+^ (%)				
Before treatment	19.56 ± 1.04	19.58 ± 1.36	0.064	0.949
3-months after treatment	18.58 ± 1.47^a^	20.56 ± 2.15^a^	4.164	<0.001
6-months after treatment	21.85 ± 2.54^a^^,^^b^	24.48 ± 1.69^a^^,^^b^	4.722	<0.001
CD8^+^ (%)				
Before treatment	21.58 ± 1.64	21.60 ± 2.01	0.042	0.966
3-months after treatment	20.97 ± 1.47^a^	22.39 ± 1.58^a^	3.604	0.001
6-months after treatment	22.56 ± 4.18^b^	24.48 ± 1.02^b^	2.444	0.018
CD4^+^/CD8^+^				
Before treatment	0.53 ± 0.01	0.55 ± 0.36	0.304	0.762
3-months after treatment	0.56 ± 0.15^b^	0.76 ± 0.45^a^	2.309	0.025
6-months after treatment	0.55 ± 0.14^b^	0.92 ± 0.25^a^^,^^b^	7.073	<0.001

Note: Compared with before treatment.

a p < 0.05; Compared with 3-months after treatment,

b p < 0.05.

### Reduction of serum inflammatory factors

Table 6 presents the comparison of patient peripheral blood cytokine levels between the Control Group and the PDT treatment group, along with corresponding *t*-values and p-values. Before treatment initiation, no significant differences were observed in IL-6, IL-8, and TNF-α levels between the Control Group and the PDT treatment group (p > 0.05). However, notable changes were evident post-treatment. At 3 months after treatment, IL-6 levels in the PDT treatment group remained relatively stable compared to the Control Group (p = 0.008). In contrast, IL-8 levels significantly decreased in the PDT treatment group compared to the Control Group (p < 0.001). TNF-α levels also showed a significant decrease in the PDT treatment group compared to the Control Group (p < 0.001). By the 6-month mark after treatment, the differences became more pronounced. IL-6 levels decreased significantly in the PDT treatment group compared to the Control Group (p = 0.001). Similarly, IL-8 levels showed a substantial decrease in the PDT treatment group compared to the Control Group (p = 0.001). TNF-α levels also exhibited a significant reduction in the PDT treatment group compared to the Control Group (p < 0.001). The results suggest that PDT treatment leads to significant reductions in IL-6, IL-8, and TNF-α levels in patient peripheral blood. These cytokines play critical roles in inflammation and immune regulation. The observed decrease in their levels post-treatment implies that PDT may effectively alleviate inflammation and immune dysregulation in patients. Further research is necessary to fully understand the mechanisms and potential therapeutic benefits of PDT-induced cytokine modulation in peripheral blood.

**Table 6:** Patient peripheral blood cytokine comparison (χ ± s).

Indicator	Control Group	PDT treatment group	*t*-value	p-value
IL-6				
Before treatment	172.37 ± 21.29	179.75 ± 12.56	1.635	0.107
3-months after treatment	175.26 ± 17.93	163.49 ± 15.34	2.732	0.008
6months after treatment	165.37 ± 8.92^a^	110.31±12.34^a^	19.806	<0.001
IL-8				
Before treatment	289.34 ± 18.28	293.47 ± 23.72	0.755	0.453
3-months after treatment	278.37 ± 27.34	124.36 ± 10.24	28.894	<0.001
6-months after treatment	276.25 ± 8.92^a^	110.31 ± 12.34^a^	59.692	<0.001
TNF-α				
Before treatment	309.22 ± 4.83	338.27 ± 17.45	8.788	<0.001
3-months after treatment	298.34 ± 9.43^a^	230.34 ± 11.26^a^	25.359	<0.001
6-months after treatment	301.36 ± 7.18^b^	108.36 ± 6.82^b^	106.748	<0.001

Note: Compared with before treatment.

a p < 0.05; Compared with 3-months after treatment,

b p < 0.05

## Discussion

HPV is a small DNA virus that can cause proliferative lesions on the skin and mucosa following infection, which can be transmitted through sexual activity or close contact, it serves as an effective indicator for screening cervical cytopathic and cervical cancer.^10^ Persistent HPV infection has been reported as one of the main risk factors for the development of precancerous lesions and cervical cancer. Currently, HC-2 is recognized as the gold standard for detecting HPV infection. In this study, 60 patients diagnosed with cervical HPV infection by HC-2 test were included to ensure result accuracy. Numerous studies have shown that over 90 % of cervical cancer patients are infected with high-risk HPV strains. As there are no specific clinical anti-HPV drugs available, traditional methods such as cervical coning may lead to complications like bleeding, uterine stenosis, and infections, thereby affecting prognosis.^11^ Therefore, early screening for HPV infection and timely safe, and effective intervention are crucial in protecting female health.

The ALA, as a novel non-surgical treatment modality, has gained extensive utilization in the management of cutaneous and mucosal tumors as well as precancerous lesions, with its remarkable efficacy and exceptional safety profile being clinically validated.^12^ The findings from this investigation demonstrate that the HPV viral clearance rates at 3-months and 6-months in the treatment group were 70.00 % and 100.00 %, respectively, which exhibit significantly superior outcomes compared to those observed in the control group (43.33 % and 80.00 %). These results indicate that The ALA effectively treats HPV infection by virtue of its distinctive mechanism: selectively absorbed and accumulated by actively proliferating HPV-infected cells due to its prerequisite role in hemoglobin synthesis; subsequent irradiation with laser of specific wavelength induces photodynamic action leading to generation of singlet oxygen and other substances that act upon infected tissues, inhibiting or eliminating activity of HPV-infected cells; thereby directly targeting diseased tissue caused by HPV infection with high specificity while preserving tissue integrity without causing evident damage.

Furthermore, the study explored the immunological response to PDT treatment by assessing the levels of CD4^+^ and CD8^+^ T-lymphocytes in cervical local tissue. The results revealed a significant increase in both CD4^+^ and CD8^+^ T-cell percentages after PDT treatment, indicating an enhanced cellular immune response. This immune activation could contribute to the clearance of HPV-infected cells and the improvement in cervical tissue health. Additionally, the study analyzed the expression levels of cytokines IL-6, IL-8, and TNF-α in the cervical tissue. The data demonstrated a substantial decrease in the levels of these pro-inflammatory cytokines at 3- and 6-months after PDT treatment. This reduction in cytokine levels indicates that PDT may effectively alleviate inflammation and immune dysregulation in the cervical region, promoting tissue healing and recovery. The principle of ALA lies in the selective absorption of light waves by normal and diseased tissues.^13^ Upon reaching the diseased tissues, non-toxic photosensitizers accumulate in tumor tissues or cells with abnormal metabolism following viral infection. Subsequently, laser irradiation at the corresponding wavelength enables the diseased tissues to absorb and generate thermal curative effects, enhance microcirculation, as well as resonate with biological radiation. This process promotes the metabolism of diseased tissue, boosts immune function, and improves nutritional status.

PDT shows promise as a potential therapeutic approach for HPV treatment, but its current limitations hinder its widespread application.^7^^,^^14^^,^^15^ PDT relies on the activation of photosensitizers by specific light wavelengths to generate reactive oxygen species that induce cell death.^16^, ^17^, ^18^ Nonetheless, the limited penetration depth of light restricts PDT's efficacy in treating deep-seated HPV lesions, reducing its application scope. Different HPV subtypes may exhibit variable responses to PDT, resulting in suboptimal treatment outcomes for specific strains. The need for personalized treatment strategies to address distinct HPV subtypes poses a significant challenge.^19^ By addressing these challenges and focusing on future research directions, PDT can be optimized to deliver effective and targeted treatment for HPV infections, improving patient outcomes and overall disease management.^20^ Continued efforts in scientific investigation and interdisciplinary collaboration will pave the way for PDT's successful integration into standard clinical practices for HPV treatment.^21^, ^22^, ^23^

The present study's findings are consistent with previous research indicating that PDT is a promising therapeutic approach for HPV infection and associated cervical lesions. The minimal adverse reactions observed during the treatment course further strengthen the safety profile of PDT as a viable treatment option.^6^^,^^24^^,^^25^ However, some limitations of the study should be acknowledged. Firstly, the sample size was relatively small, warranting larger-scale studies to validate the results. Secondly, the follow-up period was limited to 6-months; longer-term follow-ups would provide more comprehensive insights into the long-term efficacy of PDT in HPV management.

## Conclusion

Our study highlights the positive impact of PDT on cellular immune function in patients with cervical HPV infection. The findings reveal a significant increase in the proportions of CD4^+^ and CD8^+^ T-cells following PDT treatment, indicative of a strengthened cellular immune response. Additionally, PDT resulted in a noteworthy decrease in pro-inflammatory cytokines, including IL-6, IL-8, and TNF-α, further supporting its role in modulating the immune environment associated with HPV infection.

## Ethical approval

All procedures conducted in this study involving human participants adhered to the ethical standards of the institutional and/or national research committee, in line with the 1964 Helsinki Declaration and its subsequent amendments or comparable ethical guidelines. The study received approval from the 10.13039/501100004863Zhejiang Medical & Health Group Hangzhou Hospital (Approval no: Ls20240003).

## Funding

This study was supported by a research grant from Hangzhou Medical College, Project Number Y202249263.

## Declaration of competing interest

The authors declare no conflicts of interest.
